# Updates on the Risk of Neuropsychiatric and Gastrointestinal Comorbidities in Rosacea and Its Possible Relationship with the Gut–Brain–Skin Axis

**DOI:** 10.3390/ijms21228427

**Published:** 2020-11-10

**Authors:** Yu Ri Woo, Yu Jin Han, Hei Sung Kim, Sang Hyun Cho, Jeong Deuk Lee

**Affiliations:** Department of Dermatology, Incheon St. Mary’s Hospital, College of Medicine, The Catholic University of Korea, Incheon 21431, Korea; w1206@naver.com (Y.R.W.); thyme3700@gmail.com (Y.J.H.); hazelkimhoho@gmail.com (H.S.K.); drchos@yahoo.co.kr (S.H.C.)

**Keywords:** rosacea, comorbidity, psychiatric disorders, neurologic disorders, gastrointestinal disorders, risk

## Abstract

Rosacea is a common chronic cutaneous inflammatory disorder. Recently, patients with rosacea were identified as having a higher risk of developing various comorbidities such as cardiovascular disease, psychiatric disorders, neurologic disorders, and gastrointestinal disorders. However, the risks of some comorbidities in patients with rosacea are somewhat contradictory, depending upon the study design. Moreover, pathomechanisms associated with the comorbidities of patients with rosacea remain poorly elucidated. The purpose of this review was to provide the most up-to-date evidence on the risks of neuropsychiatric and gastrointestinal comorbidities in patients with rosacea. Moreover, the molecular pathomechanisms associated with neuropsychiatric and gastrointestinal comorbidities in patients with rosacea were evaluated based on recent studies. This review was also intended to focus more on the role of the gut–brain–skin axis in the association of neuropsychiatric and gastrointestinal comorbidities in rosacea.

## 1. Introduction

Rosacea is a common chronic immune-mediated inflammatory cutaneous disorder with an estimated prevalence of about 0.91–8.5% [1]. Clinically, it is characterized by chronic recurrent episodes of flushing and persistent erythema of the central face. Phymatous change, flushing, papulopustules, and telangiectasia are commonly observed cutaneous signs of rosacea. Moreover, a variety of combinations and degrees of clinical signs and symptoms of rosacea can be observed in each individual [1].

Although the exact pathogenesis of rosacea needs further elucidation, the development of the clinical manifestations of rosacea can be explained by multifactorial etiologies, including genetic predisposition, epidermal barrier disruption, dysregulation of the innate and adaptive immune system, neuroinflammation, and neurovascular dysregulation [2]. The complex pathophysiology of rosacea suggests that it is not limited to the skin but could also be associated with multiple systemic disorders. To date, several epidemiological studies have also identified the possible association between rosacea and various comorbid disorders. Specifically, the association between cardiovascular disorders, such as hypertension and coronary artery disease, and rosacea, has been confirmed in previous studies [3,4]. However, some inconsistencies in the association between rosacea and other comorbidities still exist. Therefore, this study was intended to review the most up-to-date epidemiological evidence to more clearly assess the risk of comorbid diseases in rosacea. Among a variety of comorbidities of rosacea, this study was intended to focus more on identifying the association of neurologic disorders, psychiatric disorders, gastrointestinal disorders, and rosacea. To clarify this, we further assessed the possible shared pathophysiological mechanisms of rosacea and its comorbid disorders. As a gut–brain–skin axis model has been suggested for several decades [5], and recent progress in microbiome research has solidified this hypothesis about the effect of the gut–brain–skin axis in cutaneous disorders, this review also investigated a potential association between the gut–brain–skin axis and the comorbidities of rosacea. Identifying the real burden of comorbidities in patients with rosacea might help provide a multidisciplinary approach to the patient and serve as a bridge to the unknown etiological pathogenesis of rosacea.

## 2. The Risk of Neurologic Diseases in Rosacea

Among a variety of neurologic disorders, an association between Parkinson’s disease and rosacea has long been explored. Fischer et al. [6] first observed a high rate of rosacea among patients with Parkinson’s disease in 2001. They found an 18.6% prevalence of active rosacea among patients treated for Parkinson’s disease [6]. Consequently, a nationwide study conducted in the Danish population found an increased adjusted risk of new-onset Parkinson’s disease (incidence rate ratio (IRR): 1.91; 95% confidence interval (CI): 1.52–1.92) in patients with rosacea compared with a reference population [7]. Besides, patients treated with tetracycline showed a decreased risk of Parkinson’s disease [7]. A cohort study conducted in the United States using the electronic medical records of 803,005 individuals also found an increased risk of Parkinson’s disease in patients with rosacea (odds ratio (OR): 1.39; 95% CI: 1.04–1.85) compared with the control population [8]. We summarize recent nationwide population-based cohort and case–control studies analyzing the risk of each neurologic disorder in patients with rosacea in Table 1.

Several researchers have proposed that increased matrix metalloproteinase (MMP) activity might provide the mechanistic association between Parkinson’s disease and rosacea. The increased expression of MMP-3 and MMP-9 was implicated in the mouse model of Parkinson’s disease [15,16]. In addition, increased expression of MMPs and downregulation of the inhibitors of MMPs were also observed via real-time PCR analysis in patients with rosacea [17]. Patients with granulomatous rosacea tended to have an increased expression of MMP-9 when compared with non-granulomatous rosacea by immunohistochemistry [18]. Moreover, a study by Karpouzis et al. [19] found a tachykinin receptor 3′ gene polymorphism rs3733631 in patients with rosacea. As neurokinin B, which is the endogenous ligand of TACR3, is well known to be involved in the pathogenesis of Parkinson’s disease, genetic polymorphism of TACR3 in rosacea could be one possible explanation for this association. Although further studies are needed to confirm this association, the potential pathogenic links between rosacea and Parkinson’s disease could be explained by this association.

In 1955, Davidson et al. [20] first reported Alzheimer’s disease accompanied by acne rosacea in one identical twin. A recent nationwide cohort study conducted in a Danish population observed that patients with rosacea showed an increased risk of dementia (adjusted hazard ratio (aHR): 1.28, 95% CI: 1.01–1.14) and Alzheimer’s disease (aHR: 1.25; 95% CI: 1.14–1.37) [9]. The increased risk of dementia (aHR: 1.42; 95% CI: 1.17–1.72) and Alzheimer’s disease (aHR: 1.92; 95% CI: 1.44–2.58) was also observed when they limited patients to those with rosacea diagnosed by a hospital dermatologist [9]. The plausible explanation for this association could be MMPs and antimicrobial peptides (AMPs). Increased expression of MMP-3 is also observed in the cerebrospinal fluid of patients with Alzheimer’s disease, and levels of MMP-3 are associated with the duration of the disease. Serum levels of MMP-9 are also increased in patients with Alzheimer’s disease [21]. With regard to rosacea, the increased expression of MMPs in rosacea has been shown in several studies. Moreover, treatment with doxycycline, a commonly used oral medication for rosacea, can inhibit the activity of kallikrein-related peptidases by inhibiting MMP expression [22]. The study also revealed the suppressed expression of β-amyloid and tau protein, which is a well-known pivotal protein in the pathogenesis of Alzheimer’s disease in patients with Alzheimer’s disease after treatment with tetracycline [23,24,25]. With regard to AMPs, the involvement of AMPs in the pathogenesis of rosacea has been well established [26] and β-amyloid was recently considered as a kind of antimicrobial peptide in Alzheimer’s disease [27]. The abovementioned shared pathogenic links between Alzheimer’s disease and rosacea could be the possible explanation for this association.

Another chronic neurovascular disorder, migraine, shares a variety of clinical features with rosacea. Migraine and rosacea both have features of chronic recurrent paroxysmal episodes with disabling symptoms in the trigeminal innervated area [28,29,30]. Indeed, Tan et al. [31] first reported that 44% of the patients with rosacea had experienced migraines. Thereafter, several epidemiological studies reported the association between rosacea and migraine [10,11,32,33,34]. Recently, Egeberg et al. [11] found an increased risk (aHR: 1.31; 95% CI: 1.23–1.39) of migraine in patients with rosacea. Of note, patients with phymatous rosacea showed no significant association with migraine, whereas patients with ocular rosacea showed an increased risk (aHR: 1.69; 95% CI: 1.43–1.99) of migraine [11]. A population-based study conducted in the United Kingdom observed a significant association between rosacea and migraine in females (OR: 1.22; 95% CI: 1.16–1.29) [10]. The most recent systematic review and meta-analysis also reported the possible association between the development of migraine and rosacea [35].

**Figure 1:**
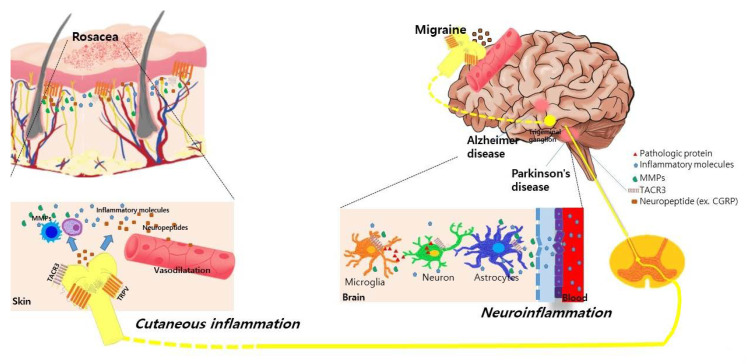
Proposed common underlying mechanisms between rosacea and various neurological disorders. Activation of transient receptor potential vanilloid type 1 (TRPV) receptors in neuronal tissues and skin induces the release of neuropeptides such as calcitonin gene-related peptide (CGRP) and substance P in patients with rosacea and migraine. These neuropeptides induce the vasodilation of cutaneous blood vessels and degranulation of mast cells, further stimulating the release of inflammatory molecules and matrix metalloproteinases (MMPs) in rosacea. In Parkinson’s disease and Alzheimer’s disease, the increased expression of MMPs is also observed in the neural tissue, cerebrospinal fluid, and serum of patients, which disrupts the blood–brain barrier and further induces neuroinflammation. Genetic polymorphisms in the TACR3 gene are found in patients with rosacea and Parkinson’s disease. This shared pathogenic link might synergistically influence the association between neurologic disorders and rosacea.

Alterations in facial blood flow and neuroinflammation could be a possible explanation for this relationship. Cutaneous facial blood flow is increased in the frontotemporal region during attacks of migraine [36]. Patients with migraine also exhibited an altered sympathetic regulation of facial microcirculation through the activation of neuropeptides due to various triggers [37,38]. In patients with rosacea, dilatation in precapillary arterioles and postcapillary venules and the subsequent disruption of the blood vessels causes the flushing and persistent erythema of rosacea. Moreover, the expression of transient receptor potential vanilloid type 1 (TRPV1) was increased both in the human scalp arteries of patients with chronic migraine [39] and in the skin of patients with rosacea [40]. Activation of the TRPV receptor induces the release of neuropeptides such as calcitonin gene-related peptide (CGRP), which is considered a pivotal pathogenetic factor in triggering both migraine and rosacea by mediating the vasodilation and degranulation of mast cells [40,41]. The proposed mechanisms explaining the association between neurologic disorders and rosacea are summarized in Figure 1.

## 3. The Risk of Psychiatric Diseases in Rosacea 

Due to the chronic nature of rosacea and facial presentation of the disease, patients with rosacea suffer from poor psychological well-being [42]. Psychological aggravating factors such as stress, anxiety, immature personality with excessive feelings of shame and guilt, and social anxiety secondary to easy blushing could worsen the flushing in patients with rosacea and be a factor involved in the vicious cycle of rosacea [12,42]. Facial erythema, which is the characteristic clinical feature of rosacea, results in more impaired health-related quality of life in patients with rosacea than its inflammatory lesions [43]. A recent study by Wu et al. [44] also found that patients with rosacea showed increased dermatology life quality index scores and hospital anxiety and depression scale scores than controls, suggesting the significant negative psychological impact of rosacea on the Chinese patients in the study. The increased psychosocial burden associated with rosacea has caused many researchers to analyze the risk of psychiatric comorbid disease in rosacea. Table 1 summarizes recent nationwide population-based cohort and case–control studies analyzing the risk of different psychiatric disorders in patients with rosacea.

A case–control study conducted in the United States reported that patients with rosacea had an increased risk of depressive disease (OR: 4.81; 95% CI: 1.39–16.62) compared with the controls [12]. In a nationwide study conducted in a Danish population, Egeberg et al. [13] also found that the patients with both mild and moderate to severe rosacea showed an increased risk of depression (mild rosacea, IRR: 1.89; 95% CI: 1.82–1.96; moderate to severe rosacea, IRR: 2.04; 95% CI: 1.96–2.12). A more recent study found that patients with rosacea had an increased risk of overall psychiatric disorders (adjusted hazard ratio (aHR): 2.76, 95% CI: 2.65–2.87) after adjusting for age, sex, comorbidity, and residence [14]. Among a variety of psychiatric disorders, the highest risk of psychiatric comorbidities was for phobic disorder (aHR: 7.84; 95% CI: 7.52–8.17) followed by obsessive-compulsive disorder (aHR: 6.38; 95% CI: 6.13–6.65), major depressive disorder (aHR: 3.78; 95% CI: 3.63–3.94), bipolar disorder (aHR: 3.06; 95% CI: 3.06–3.32), and anxiety (aHR: 2.91; 95% CI: 2.79–3.03) [14]. The increased risk of anxiety disorder was proposed in a study by Incel Uysal et al. [45]. Female patients with rosacea showed a higher risk of having generalized anxiety disorder (OR: 2.8; 95% CI: 1.15–7.37) than males [45]. The study examined a US national inpatients sample and found that patients diagnosed with rosacea showed increased odds (OR: 1.70; 95% CI: 1.56–1.95) of a primary admission for mental health disorders [46]. Treatment for rosacea, particularly for blushing, might help improve depressive symptoms and social anxiety [47]. Similarly, the effective management of rosacea symptoms results in a significant improvement in health-related quality of life.

The plausible explanation for this association could be abnormalities in the gut–brain axis and its shared inflammatory pathways. Altered gastrointestinal microbiota was also frequently observed in psychological disorders including depression and anxiety disorders [48,49,50]. Psychological stressors are known to induce the production of various neurotransmitters or the release of neuropeptides from nearby enteroendocrine cells [51]. This could increase the permeability of the intestine and consequently cause intestinal and systemic inflammation. Indeed, an increased prevalence of infection with *Helicobacter pylori (H. pylori*) and small intestinal bacterial overgrowth and abnormalities in skin microbiota were observed in patients with rosacea [48,52,53]. Based on these findings, we can suppose a possible association between rosacea and psychiatric disorders via the gut–brain–skin axis [14,54]. IL-17 has been implicated as a key cytokine in central nervous system diseases and mediates psychiatric disorders. Indeed, Th17 cells are increased in the blood of patients with depression and promote depression-like behaviors in mice. Similarly, IL-17 plays an important role in the development and aggravation of rosacea [55]. However, when compared with other chronic cutaneous skin diseases including acne, atopic dermatitis, and psoriasis, the psychosocial aspects of rosacea have been underestimated and further research is needed to properly assess the risk of psychological burden in patients with rosacea.

## 4. The Risk of Gastrointestinal Disorders in Rosacea

In 1965, Watson et al. [56] reported a case of small bowel disease in a patient with rosacea. Since then, various epidemiological studies have reported the association between gastrointestinal disorders and rosacea.

*H. pylori* is a Gram-negative bacterium that plays a pivotal role in the development of gastritis, peptic ulcers, and gastric cancer. In addition to its association with various gastrointestinal disorders, an increased prevalence of *H. pylori* infection in patients with rosacea was also reported. About 88% of the patients with rosacea were infected with *H. pylori,* compared with 65% of the non-ulcer dyspeptic controls [57]. Moreover, eradication of *H. pylori* improved the skin symptoms of rosacea [57,58,59]. However, a Danish population-based cohort study reported no significant association between *H. pylori* infection (hazard ratio (HR): 1.04; 95% CI: 0.96–1.13) in patients with rosacea compared with control subjects [60]. Although they also found an increased prevalence of *H. pylori* infection in patients with rosacea, the prevalence of new-onset *H. pylori* infection was not increased in patients with rosacea, suggesting that *H. pylori* infection could be one of the predisposing factors in rosacea by acting as a source for the growth of other intestinal bacteria [60]. A recent systematic review and meta-analysis identifying the risk of *H. pylori* infection in rosacea concluded that there was a weak but non-significant association with rosacea [61]. However, the authors suggested that studies conducted with the C-urea breath test, which has a higher diagnostic value than the serologic test for *H. pylori* infections, showed a strong association with rosacea compared with studies conducted with serology tests, implying that differences in the diagnostic method for *H. pylori* infections in previous studies might contribute to these differences [61]. In addition, different strains of *H. pylori* possess specific virulence factors such as cytotoxin-associated gene A (CagA), which could contribute to the difference in the study results [62].

The proposed mechanisms explaining this association include the various cytotoxins and reactive oxygen species produced by *H. pylori*, which result in inflammation of the gastric mucosa and cutaneous inflammation via the increased production of inflammatory mediators, including prostaglandins, leukotrienes, vasoactive histamines, and cytokines, and the infiltration of inflammatory cells including monocytes, neutrophils, and lymphocytes [63].

Among a variety of gastrointestinal disorders, a high prevalence of celiac disease (HR: 1.46; 95% CI: 1.11–1.93), Crohn’s disease (CD) (HR: 1.45; 95% CI: 1.19–1.77), ulcerative colitis (UC) (HR: 1.19; 95% CI: 1.02–1.39), and inflammatory bowel syndrome (HR: 1.34; 95% CI: 1.19–1.50) was observed in patients with rosacea compared with a control population in a large population-based cohort study conducted in Denmark [60] (Table 2). Recent evidence supports an association between rosacea and inflammatory bowel disease (IBD). A large population-based case–control study using a UK-based clinical practice research datalink found that a history of UC was associated with an increased risk of rosacea (OR: 1.65; 95% CI: 1.43–1.90) [64]. A history of CD was also associated with an increased risk of rosacea (OR: 1.49; 95% CI: 1.25–1.77) [64]. A cross-sectional study conducted in US female nurses reported that patients with rosacea had an increased risk of CD (HR: 2.20; 95% CI: 1.15-4.18). However, the study found no significant association between rosacea and UC [65]. A study by Kim et al. [66] also reported that patients with IBD had an increased risk of rosacea (OR: 2.17; 95% CI: 1.59–2.96) compared with control subjects. They also reported that male patients with IBD had a greater risk of rosacea than female patients [66]. Recently, a meta-analysis was conducted to examine the association between rosacea with IBD [67]. A meta-analysis of case–control studies found a significantly increased risk of UC (OR: 1.64; 95% CI: 1.43–1.89) in patients with rosacea. A meta-analysis of cohort studies revealed an increased risk of CD (HR: 1.58; 95% CI: 1.14–2.20) and UC (HR: 1.18; 95% CI: 1.01–1.37) in patients with rosacea [67].

The association between IBD and rosacea could be explained by alterations in both innate and adaptive immunity. The activation of mast cells, macrophages, and toll-like receptor 2 and production of reactive oxygen species, MMP, tumor necrosis factor, and interleukin(IL)-1β are known to be involved in the inflammatory pathogenesis of both rosacea and IBD [2,68]. With regards to adaptive immunity, inflammation induced by T helper (Th)1 cells, Th17 cells, and B cells contribute both to the pathogenesis of rosacea and IBD via the release of TNF, IL-17, and interferon-gamma [2,68]. Finally, shared genetic risk loci between rosacea and IBD such as HLA-DRB1*03:01 and butyrophilin-like 2 (BTNL2) could be another explanation for this association. Moreover, an increased prevalence of the glutathione S-transferases theta 1 (GSTT1) null genotype was observed in patients with rosacea and IBD [69,70].

In addition to IBD, studies have identified the possible association between rosacea and other gastrointestinal disorders. An increased prevalence of small intestinal bacterial overgrowth (SIBO) was observed in patients with rosacea. A prospective randomized controlled study found that 46% of the patients with rosacea had SIBO [71]. Moreover, after treatment with rifaxamin, clearance of the skin lesions in patients with rosacea was observed in 20 of 28 patients [71]. Another study by Weinstock et al. [52] found an increased risk of SIBO in patients with rosacea (relative risk (RR): 2.1; 95% CI: 1.7–15.1) compared with the controls. Among rosacea patients with SIBO prescribed rifaximin, 46% of the patients with rosacea reported cleared or marked improvement in their skin lesions [52]. The suggested pathomechanism involved in the association between rosacea and SIBO could be explained by increases in shared circulating cytokines, especially tumor necrosis factor-alpha [72]. However, a Danish population-based cohort study found no significant association between SIBO (HR: 0.71; 95% CI: 0.18–1.86) in patients with rosacea compared with control subjects [60]. Although there are some contradictory results with regards to the association between rosacea and SIBO, studies have reported the high prevalence of SIBO in patients with IBD compared with controls [73,74], supporting the potential association between SIBO, IBD, and rosacea.

Dysregulation of the skin and gut microbial community has long been considered to have a pivotal role in rosacea and gastrointestinal disorders. Although related microbial species such as *Demodex folliculorum, Staphylococcus epidermidis, H. pylori, Bacillus oleronius*, or *Chlamydia pneumoniae* have not been shown to directly cause the disease, dysbiosis in the composition of the skin and gut microbiota is suspected to play a major role in the pathogenesis of both rosacea and gastrointestinal disorders via impairing the innate and adaptive immunity of the skin and gut epithelium [75]. Intestinal bacteria could also affect the activation of the plasma kallikrein–kinin system (PKKS) [76]. The activation of PKKS is observed in patients with intestinal inflammation and rosacea [77,78] and this shared pathogenic link could be a possible explanation for the association.

## 5. Conclusions

Rosacea is considered a chronic recurrent systemic inflammatory disorder. Several studies have analyzed the risk of comorbidities in rosacea. The knowledge presented in this review suggests the existence of links between rosacea and various neurologic disorders, psychiatric disorders, and gastrointestinal disorders.

A growing body of evidence has reported the pathogenic factors of rosacea. The interplay of factors such as genetic predisposition, triggering factors, immune alterations, neuroinflammation, and neurovascular dysregulation has been suggested to play a pivotal role in the pathophysiology of rosacea. The possible association of diverse comorbidities in rosacea could also be explained by the shared pathogenic pathways and molecules involved both in comorbid disorders and rosacea.

**Figure 2:**
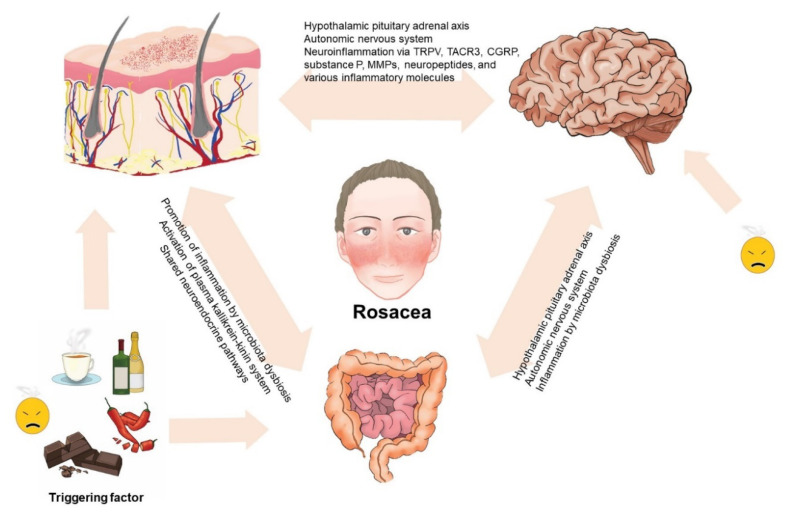
Proposed role of the gut–brain–skin axis in rosacea. The complex interplay among the skin, gut, and brain sustains an intricate balance. Disturbances in the balance might influence the function of the skin, gut, and brain and the occurrence of rosacea and its mental, psychiatric, and gastrointestinal comorbidities.

Some triggering factors such as stress and diet are considered to have a major role in the exacerbation of rosacea. For example, alcoholic beverages, spicy food, hot drinks, and chocolate are well-known dietary triggers of rosacea. Diet has a substantial impact on the regulation of the human microbiome [72] and dysbiosis in the intestine has a negative impact on skin function. Metabolites of the microbiota could accumulate in the skin and thereafter damage the integrity of the skin barrier and promote further inflammation. Whether microbiota could be considered causative agents or innocent bystanders in the development of rosacea needs further elucidation [80]. Studies have supported the concept of imbalances in the diverse microbiota in the skin and gut of patients with rosacea. Moreover, as the skin and gut both have neuroendocrine pathways, this could also affect the function of the brain. The association with the gut–brain axis has also been established via multiple parallel pathways between them, including the autonomic nervous system and the pituitary–hypothalamus–adrenal axis [81]. The proposed role of the gut–brain–skin axis in rosacea is depicted in Figure 2.

The findings from this study present the concept of the involvement of the gut–brain–skin axis in rosacea. While diverse studies support this association, further experimental studies are needed to more clearly identify the exact pathophysicological mechanisms of this axis and its association with rosacea pathogenesis. In clinical practice, it is necessary to manage the stress of patients with rosacea and to strictly control the intake of some foods that can lead to the imbalance of the gut–brain–skin axis in rosacea. In addition, probiotic supplementation in patients with rosacea might help in improvement of the gut environment. As antibiotic treatment resulted in the improvement of some neurologic and gastrointestinal disorders, which were also associated with rosacea, we suspect that antibiotic therapy could be a promising option in controlling the altered gut–brain–skin axis of patients with rosacea.

In addition to the abovementioned comorbidities, further identification of comorbidities in patients with rosacea is needed in the future. The risk factors of each comorbidity in patients with rosacea also need to be further explored. Further, as increased risks of comorbidities are frequently observed in patients with rosacea, clinicians should always examine the possible presence of comorbidities in patients with rosacea to provide a multidisciplinary approach to treating the comorbidities.

## Figures and Tables

**Table 1:** Summary of recent (2000–2020) population-based cohort studies investigating the risk of psychiatric and neurologic disorders in patients with rosacea.

Disorder	Study	Study Population	Relative Risk of Measures
**Neurologic disorder**	Egeberg (2016) [7]	Rosacea: 68,053 Control: 5,404,692	Parkinson’s disease aIRR 1.71 (1.52–1.92)
	Mathieu (2018) [8]	Rosacea: 14,696 Control: 399,383	Parkinson’s disease OR 1.39 (1.04–1.85)
	Egeberg (2016) [9]	Rosacea: 82,439 Control: 5,509,279	Dementia aHR 1.28 (1.01–1.14); Alzheimer’s disease aHR 1.25 (1.14–1.37)
	Spoendlin (2013) [10]	Rosacea: 53,927 Control: 53,927	Migraine in women aOR 1.22 (1.16–1.29)
	Egeberg (2016) [11]	Rosacea: 49,475 Control: 4,312,213	Migraine aHR 1.31 (1.23–1.39); migraine in ocular rosacea aHR 1.69 (1.43–1.99)
**Psychiatric disorder**	Gupta (2005) [12]	Rosacea visits: 13,978,704 Control: 594,766,021	Depressive disease OR 4.81 (1.39–16.62)
	Egeberg (2016) [13]	Rosacea: 55,437 (Mild: 30,725; moderate to severe: 24,712) Control: 4,576,904	Depression IRR in mild rosacea 1.89 (1.82–1.96); in moderate to severe rosacea 2.04 (1.96–2.12) Anxiety disorder IRR in mild rosacea 1.80 (1.75–1.86); in moderate to severe rosacea 1.98 (1.91–2.05)
	Hung (2018) [14]	Rosacea: 7881 Control: 31,524	Total psychiatric disorders aHR 2.76 (2.65–2.87); phobic disorder aHR 7.84 (7.52–8.17); obsessive-compulsive disorder aHR 7.84 (7.52–8.17); major depressive disorder aHR 3.78 (3.63–3.94); bipolar disorder aHR 3.19 (3.06–3.32); anxiety aHR 2.91 (2.79–3.03); personality disorder aHR 2.85 (2.73–2.97); manic disorder aHR 2.63 (2.52–2.74); schizophrenia aHR 2.28 (2.19–2.38); attention deficit hyperactivity disorder aHR 1.04 (1.00–1.08)

Abbreviations: RR, relative risk; OR, odds ratio; HR, hazard ratio; IRR, incidence rate ratio. The values in brackets indicate the 95% confidence interval.

**Table 2:** Recent cohort, cross-sectional, and case–control studies (2010–2020) identifying an associated risk between inflammatory bowel disease and rosacea.

Study	Study Design	Study Population	Main Outcomes
Spoendlin (2016) [64]	Case–control	Rosacea: 80,957 Control: 80,957	A history of UC is associated risk of rosacea (OR: 1.65; 95% CI: 1.43–190); a history of CD is associated with risk of rosacea (OR: 1.49; 95% CI: 1.25–1.77)
Li (2016) [65]	Cross-sectional	Rosacea: 1127 female nurses; Control: 95,187 female nurses	No association with UC CD (HR: 2.20; 95% CI: 1.15–4.18)
Egeberg (2017) [60]	Cohort	Rosacea: 49,475 Control: 4,312,213	Celiac disease (HR: 1.46; 95% CI: 1.11–1.93); Crohn’s disease (CD) (HR: 1.45; 95% CI: 1.19–1.77); ulcerative colitis (UC) (HR: 1.19; 95% CI: 1.02–1.39); inflammatory bowel syndrome (HR: 1.34; 95% CI: 1.19–1.50) No association with SIBO and *H. pylori* infection
Wu (2017) [79]	Cohort	Rosacea: 89,356 Control: 178,712	IBD (HR: 1.94; 95% CI: 1.04–3.63)
Kim (2018) [66]	Cross-sectional	IBD: 40,843 (CD: 12,646; UC: 28,197) Control: 122,529	Rosacea (OR: 2.173; 95% CI: 1.590–2.969); rosacea risk among UC patients (OR: 1.979; 95% CI: 1.389–2.819); rosacea risk among CD patients (OR: 2.735; 95% CI: 1.708–4.380)

Abbreviations: CD, Crohn’s disease; HR, hazard ratio; IRR, incidence rate ratio; SIBO, small intestinal bacterial overgrowth; OR, odds ratio; RR, relative risk; UC, ulcerative colitis. Values in brackets indicate the 95% confidence interval.

## Abbreviations

AMP	antimicrobial peptide
CD	Crohn’s disease
CGRP	calcitonin gene-related peptide
CI	confidence interval
HR	hazard ratio
IBD	inflammatory bowel disease
IL	interleukin
IRR	incidence rate ratio
MMP	matrix metalloproteinase
OR	odds ratio
PKKS	plasma kallikrein–kinin system
RR	relative risk
SIBO	small intestinal bacterial overgrowth
Th	T helper
TRPV	transient receptor potential vanilloid type
UC	ulcerative colitis
